# Examining gender and ethnic disparities in scientific authorship to promote a culture of equity, diversity and inclusion at a university school of public health

**DOI:** 10.1098/rspb.2025.0313

**Published:** 2025-11-26

**Authors:** Paula Christen, Julia Michalow, Tristan Naidoo, Hillary Topazian, Sabine L. van Elsland, Abeer M. Arif, Marc Baguelin, Gemma Clunie, Sarah Essilfie-Quaye, Daniela Fecht, Tini Garske, Sondus Hassounah, Jenny Husbands, Wendy Kwok, Sequoia Leuba, Clare McCormack, Kate M. Mitchell, Matteo Pianella, Michael Pickles, Shazia N Ruybal-Pesántez, Nora Schmit, Chinedu Udeh-Momoh, Anne Cori, Isobel Blake, Lucy C. Okell

**Affiliations:** ^1^ Center for Epidemiological Modelling and Analytics, University of Nairobi, Nairobi, Kenya; ^2^ MRC Centre for Global Infectious Disease Analysis, School of Public Health, Imperial College London, London, UK; ^3^ Abdul Latif Jameel Institute for Disease and Emergency Analytics, Imperial College London, London, UK; ^4^ Department of Epidemiology and Biostatistics, Imperial College London, London, UK; ^5^ Department of Surgery and Cancer, Imperial College London, London, UK; ^6^ School of Public Health, Imperial College London, London, UK; ^7^ EQuity Laboratory, Imperial College London, London, UK; ^8^ MRC Centre for Environment and Health, Imperial College London, London, UK; ^9^ MRC Centre for Outbreak Analysis and Modelling, Infectious Disease Epidemiology, Imperial College London, London, UK; ^10^ Department of Nursing and Community Health, Glasgow Caledonian University London, London, UK; ^11^ Institutet för Internationell Ekonomi, Stockholm University, Stockholm, Stockholm County, Sweden; ^12^ Instituto de Microbiología, Universidad San Francisco de Quito, Quito, Ecuador; ^13^ School of Medicine, Wake Forest University, Winston-Salem, North Carolina, USA; ^14^ Aga Khan University, Nairobi, Kenya; ^15^ Brain and Mind Institute, Imperial College London, London, UK

**Keywords:** research culture, equity, diversity and inclusion, research disparities

## Abstract

In public health research, diverse perspectives are vital to identify biases that homogenous teams might miss. Since publication metrics influence career progression, we investigated publication rate disparities within a School of Public Health. We analysed 18 322 peer-reviewed publications by 513 affiliated researchers between 2014 and 2023 using multivariable regression models and network analysis to assess the impact of gender, ethnicity, job level and centrality in the School’s research network on publication rates. We found a persistent gender gap in publication rates across job levels and ethnicities, with men publishing more than women (incidence rate ratio 1.30, 95% confidence interval (CI): 1.15–1.46). This disparity was present from early career levels and amplified in senior roles, where men were over-represented (71.2% of men at Professor level). Unadjusted analyses indicated higher publication rates for white researchers (median of one publication more per person per year). The COVID-19 pandemic led to increased publication rates for both genders, but the gender gap persisted, with men publishing 1.27 (95% CI: 1.10–1.46) times more than women in 2020/2021. This study underscores the need to identify and address root causes of these disparities to foster an inclusive research environment where diverse contributions are recognized and valued.

## 1. Background

Reasons for fostering equity, diversity and inclusion (EDI) in the workplace are vast. In public health research, diverse perspectives enhance the ability to identify biases that homogenous teams would otherwise overlook [1,2]. Increasing diversity within research teams can enhance the quality of research and help address the unique public health challenges faced by different population subgroups [3]. Beyond this, we need to act on the systemic issues in education, academia and the scientific publishing community that have led to inequities and unequal opportunities.

Despite progress driven by EDI initiatives such as the Athena Swan Charter [4] (established 2005) and Race Equality Charter in the United Kingdom (UK) [5] (established 2015), evidenced by high entry rates for pupils from Chinese, Indian and Black African backgrounds into higher education [6], inequities persist across the educational and publishing landscape. Growing concerns about boys being left behind in school are mirrored in higher education [7], where women now outnumber men in enrolment [8]. Yet, women remain under-represented in the upper echelons of academia, which are dominated by white men [9]. This phenomenon, often referred to as the ‘leaky pipeline’ [9] or ‘scissors effect’ [10], is well-documented across many disciplines and countries [11–15].

While the importance of understanding representation in academia is well established, the position of minority ethnic groups in academia has received little attention [16]. In the United States (US), women from minoritized groups are significantly under-represented in science, technology, engineering and mathematics (STEM) faculties compared to their share of the population, and this disparity worsens at higher academic levels [17,18]. Faculty diversity is vital, as it can significantly reduce performance gaps between students of different racial and ethnic backgrounds [17]. Moreover, a significant proportion of minority students [19] as well as faculty members [20] report experiencing discrimination or harassment, highlighting the urgent need to address systemic inequities in academia.

Gender bias has been identified in teaching ratings and salaries [21], two critical factors for retaining staff in academia [22,23]. However, contrary to omnipresent claims in the literature of sexism in the tenure-track academy [24–28], a review of the literature on gender bias in academia from 2000 to 2020 across multiple faculties in institutions across the US [21] surprisingly concludes that, while there is lower representation of women across job categories in mathematically intensive fields, overall tenure-track women are on par with tenure-track men in grant funding and journal acceptance rates. Yet, these findings vary across disciplines and may differ globally [29–32]. Few studies have accounted for observed productivity, which can moderate the tenure-track evaluation contexts.

Publication count, an incomplete quantification of productivity, is arguably a main currency of the academic profession, and a key metric considered in promotions [33–35]. This presumption that quantity is representative of quality is inherently flawed [36,37] and lacks the nuance that the reality of productivity is more broad. It encompasses not only traditional tangible research output (such as publication count) but also time devoted to teaching, community engagement and ‘academic housework’—activities often disproportionately undertaken by women [38] yet rarely formally acknowledged in promotion processes [39,40]. Nygaard *et al.* demonstrated the disproportionate benefits men receive from using publication output and impact as primary productivity indicators [41]. The need for a more comprehensive measure of productivity in academia has been recognized [42], but there are no standard guidelines on accounting for the full array of activities to allow for comparable promotion processes across institutions or even faculties [34].

Research exploring publication disparities in academia, especially those related to ethnicity, race and the intersectionality of these factors with gender, remains limited. Existing studies have revealed concerning trends: Black and Hispanic medical school graduates in the US publish significantly less than their Asian and White counterparts [43], while large-scale analyses have identified persistent gender gaps in publication count and first and last authorship [32,44–46], though some evidence suggests these may be decreasing [29,45]. However, few studies have comprehensively examined these biases across various job levels within faculty departments or isolated the specific impacts of ethnicity and race, particularly when interconnected with gender. Much of the existing work focuses on authorship within journals rather than the broader context of departmental productivity. In addition, most work that tackles the productivity causality puzzle focuses solely on the US [21], specific medical domains/sub-fields [47] or compares multiple disciplines [41,48].

We have conducted research to advance this topic by focusing on the field of public health and examining the intersection of gender and ethnicity in the context of publication productivity in the UK. We sought to understand whether publication rates contribute to the leaky pipeline, resulting in gender and ethnic disparity in our department, the School of Public Health (SPH), Imperial College London (ICL). Supported by a broader working group, our core writing team comprises^1^ and represents views from PhD student to Reader level. Ultimately, this study adds to a growing body of evidence advocating for tailored evaluation methods that acknowledge the diverse research practices and publication norms across different fields and regions, promoting a more equitable academic environment. In this commentary, we provide an overview of our findings, discuss potential explanations for the findings and propose avenues for fostering a more equitable and inclusive environment within similar departments.

## 2. Methods

We conducted a quantitative analysis of peer-reviewed papers published by researchers within ICL SPH between 2014 and 2023. This department comprises researchers in Epidemiology and Biostatistics, Infectious Disease Epidemiology, Primary Care and Public Health, the Imperial Clinical Trials Unit, the Ageing Epidemiology Research Unit and the Environmental Research Group. We quantified the publication rate by gender and ethnicity over time, adjusting for job level. Publications were obtained from Symplectic Elements [49] (the research information management system used by ICL), and gender was assigned using an algorithm based on historical name datasets and manual review [50]. We contrast ‘man’ and ‘woman’ in this piece owing to lack of data on gender identity, but acknowledge the existence of non-binary, intersex and other diverse gender identities. Job categories were scraped from ICL staff webpages. As information on promotion dates were not readily available, we relied on the assumption that the most recent SPH job title was an individual’s job category across all publication records. Ethnicity was assigned using probabilistic estimates based on full names and US Census data [51]. The number of individuals estimated to be from different ethnicities was low such that we categorized White individuals as ‘non-minoritized’ and Asian, Black and Hispanic individuals as ‘minoritized’ to ensure individual privacy. While this ethnicity categorization is likely to be inaccurate in many cases, studies have shown that substantial unconscious bias can result from a name alone (e.g., in controlled recruitment studies based on resumes) [52]. Therefore, we considered that ‘perceived ethnicity’ may still be a useful indicator of potential for bias, for example from journal editors or reviewers when a researcher submits their paper—even if it does not always reflect a person’s lived experience at work.

Negative binomial mixed-effects regression models were used to assess the effects of gender (man, woman), ethnicity (minoritized, non-minoritized), job category (Student/Research Assistant, Research Associate, Research Fellow, Lecturer, Senior Lecturer/Reader, Professor, other) and time period (2014/2015, …, 2022/2023) on annual number of publications per person, using random intercepts for each person. A network analysis was performed to quantify author centrality, measured in number of internal co-authors per year, within the SPH’s publication network. A more detailed account of the methods is presented in the electronic supplementary material.

## 3. Results

**Figure 1.**
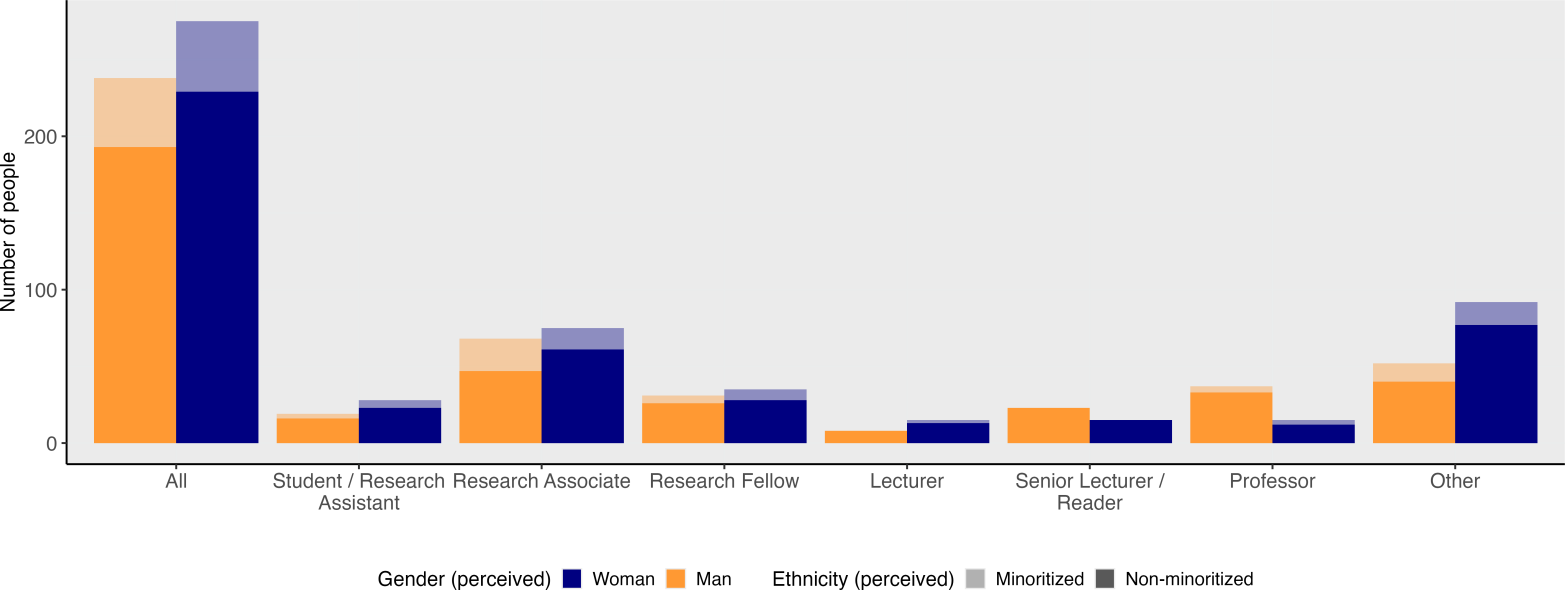
Proportion of men and women researchers by job category and perceived ethnicity group at the School of Public Health, Imperial College London, 2014–2023. See electronic supplementary material for more information on ‘other’.

**Figure 2.**
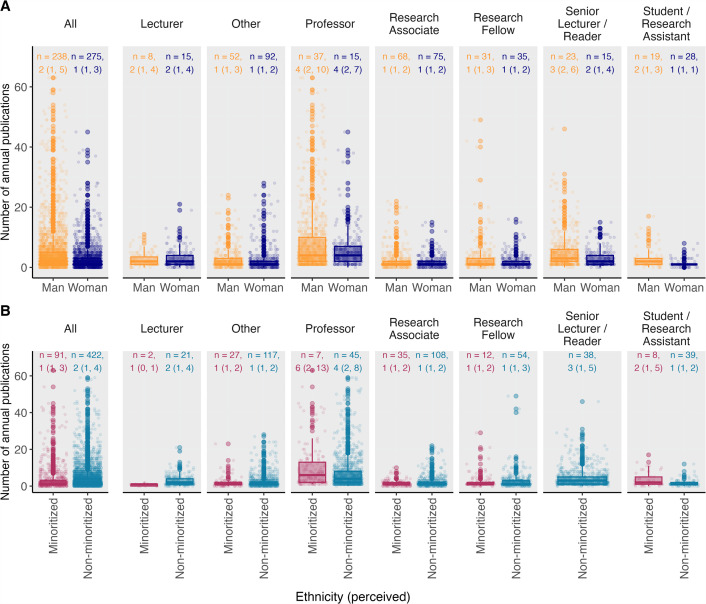
Mean number of annual publications by job category, ethnicity (perceived) and gender (perceived). The box represents the median and interquartile range (IQR) of mean number of annual publications. The boxplot whiskers represent the adjacent values, which are by convention within 1.5 times the IQR. The dots represent mean number of annual publications outside of the adjacent values, known as outliers. *n*, number of observations. Annotation on graph: median (IQR). See electronic supplementary material for more information on ‘other’.

To give a better sense of SPH, among the 275 women and 238 men researchers affiliated to the SPH between 2014 and 2023, there are more women than men from Student/Research Assistant (59.6%–40.4%) to Lecturer level (65.2%–34.8%), but men outnumber women in all more senior job categories (Senior Lecturers/Readers (39.5%–60.5%), Professors (28.8%–71.2%); figure 1). We observe a persistent gender gap in publication rates when analysing 18 322 papers by researchers affiliated to SPH between 2014 and 2023 (figure 2). Overall men publish a median of two (interquartile range (IQR): 1–4) papers per person per year, while women publish a median of one (IQR: 1–3) paper per person per year. This difference is more pronounced in senior job categories (Lecturers and Professors; electronic supplementary material, table S1). Using unadjusted incidence rate ratios (IRR), we observe that, over the study period, men in junior positions achieved the same number of publications as women 4.27 years sooner, and 4.74 years sooner for men compared to women in senior positions.

We further investigated the difference in publication rates by adjusting for job category, ethnicity and time period. We observed that men publish 1.30 (95% confidence interval (CI): 1.15–1.46) times more papers per person annually than women overall, and 1.31 (95% CI: 1.12–1.53) times more first or last author papers (electronic supplementary material; table S1). The higher number of men at more senior job levels did not explain the difference in publication rates, with male Students/Research Assistants, Research Associates and Fellows publishing between 1.43 (95% CI: 0.89–2.29) and 1.34 (95% CI: 0.99–1.83), respectively, more papers than their women counterparts. Men Senior Lecturers and Readers publish 1.68 (95% CI: 1.16–2.41) times more papers per person annually than their women counterparts. This trend persists among minoritized and non-minoritized researchers, with men consistently publishing more in both groups (electronic supplementary material; table S2). The number of internal collaborations does not explain differences in publication rates by gender. Men, compared to women, had a similar average number of co-authors within the SPH (incidence rate ratio (IRR) = 1.01, 95% CI: 0.82–1.25).

Between 2014 and 2023, annual publication rates per researcher increased, especially during the COVID-19 pandemic, i.e. from 2020 onwards. In 2020 and 2021, men and women published 1.17 (95% CI: 1.08–1.28) times more papers per person overall than in 2014 and 2015. For women, this trend picked up from 2018 onwards, while mens’ annual publication rates increased from 2014 to 2019 and then decreased. Nonetheless, men continuously published significantly more than women from 1.29 (95% CI: 1.08–1.54) times more in 2014 and 2015 to 1.58 (95% CI: 1.35–1.86) times more in 2018 and 2019 (electronic supplementary material; table S3). This gap reduced with men publishing 1.27 (95% CI: 1.10–1.46) and 1.07 (95% CI: 0.92–1.25) times more than women in 2020 and 2021, and 2022 and 2023, respectively.

**Figure 3.**
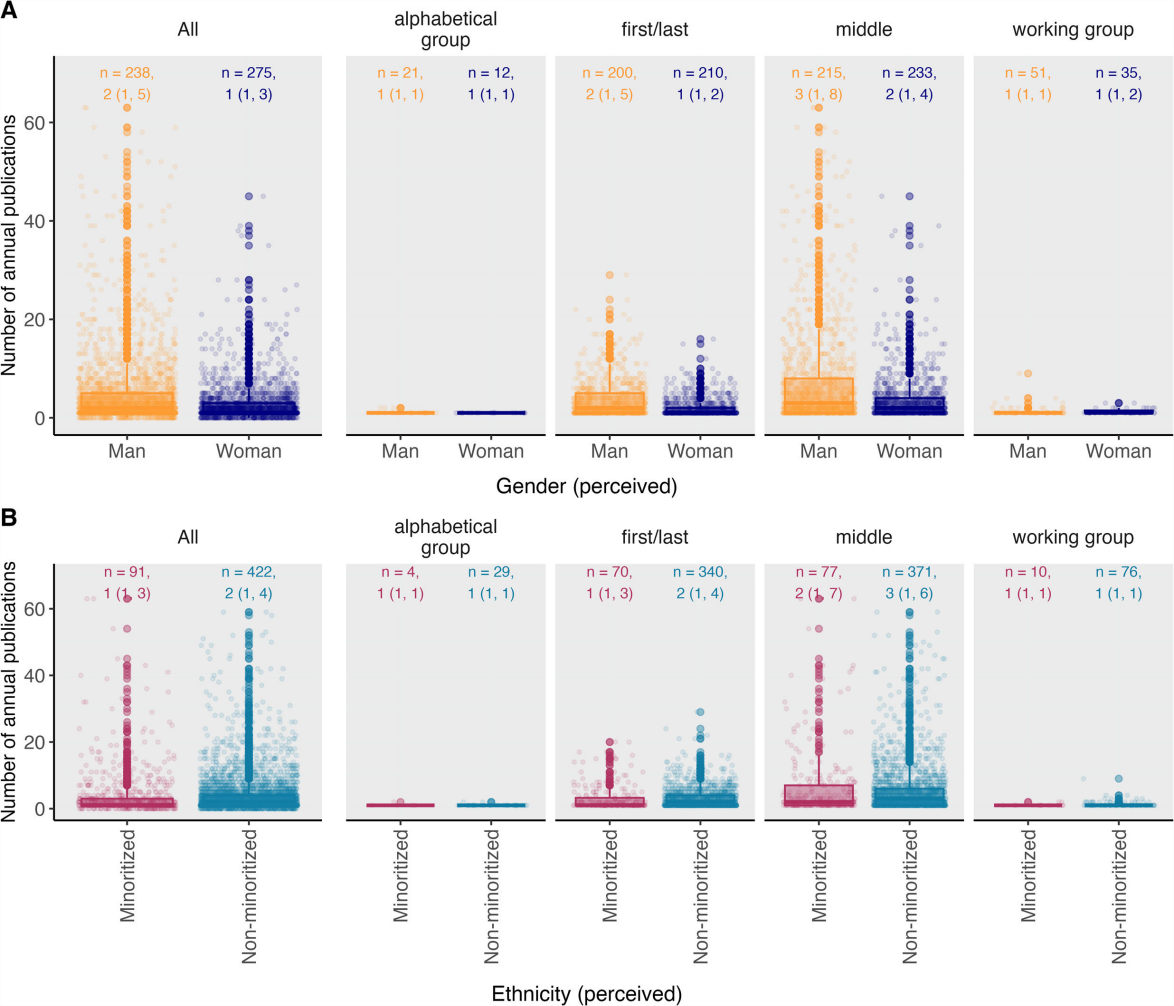
Mean number of annual publications by authorship position, ethnicity (perceived) and gender (perceived). The box represents the median and interquartile range (IQR) of mean number of annual publications. The boxplot whiskers represent the adjacent values, which are by convention within 1.5 times the IQR. The dots represent mean number of annual publications outside of the adjacent values, known as outliers. *n* = number of observations. Annotation on graph: median (IQR).

Most researchers (82.3%) in SPH were classified as White by the ethnicity algorithm, although human resource data suggest the algorithm underestimates the proportion of minoritized staff based on name. The proportion in the ethnically minoritized group is consistent across all job levels and genders (figure 1). In the unadjusted analysis, researchers from minoritized groups published a median of one (IQR: 1–3) paper per person per year, whereas researchers from non-minoritized groups published a median of two (IQR: 1–3) papers per person per year (figure 3). In the adjusted analysis, we found no statistically significant difference in overall publication rates between researchers from minoritized and non-minoritized groups. However, among women, there was a marginally lower publication rate among ethnic minorities (minoritized women published 0.79 as many papers as non-minoritized women; 95% CI 0.63–1.00).

Men researchers from minoritized groups published significantly less as first or last authors compared to their men counterparts in non-minoritized groups (IRR = 0.83, 95% CI: 0.70– 0.98), with a similar though non-significant trend among women researchers from minoritized groups (IRR = 0.79, 95% CI: 0.57–1.09). Authors from non-minoritized groups had a higher number of internal co-authors than researchers from minoritized groups in SPH (IRR = 1.81, 95% CI: 1.35–2.42).

## 4. Discussion

We found a persistent gender gap in publication rates, with men publishing significantly more than women even after adjusting for job category, ethnicity and time. These findings suggest that the higher publication rates and higher rates of first/last authorship among men researchers probably contribute to their over-representation in senior positions within SPH, since publication is a criterion for promotion.

Differences in publication output are often attributed to gender-specific parenthood roles, which disproportionately impact a women’s career duration through factors such as maternity and part-time work [53,54]. We hypothesized that these differences would manifest more prominently in senior positions, where career interruptions owing to parenthood are more likely [53]. After adjusting for differences in career length and job seniority, we found that men reached equivalent publication counts notably earlier than women: 4.27 years sooner in junior positions and 4.74 years sooner in senior positions. Assuming equal productivity during periods of full-time work, the observed gap appears too large to be explained by these factors alone [55].

Our findings resonate with observations in Norway [41,56] and Canada [57], where multidisciplinary studies revealed men publish 20%–40% more than women, highlighting that publication bias is a pervasive, potentially global issue demanding more attention. The gravity and breadth of this problem are underscored by the Declaration on Research Assessment (which ICL has committed to since 2017) and the Leiden Manifesto, guidelines and recommendations crystallized at conferences held in 2012 and 2014 [58], respectively, which explicitly advise against using quantitative evidence on publication counts and impact only for individual researcher evaluations.

However, productivity in terms of publication count does not necessarily reflect the time spent conducting the research or research engagement [59] nor does it necessarily correlate with research complexity or research quality. Alternative metrics considered for promotions in research can be determined through a bibliometric approach (e.g. citation counts, citation rates, h-index); while these more accurately measure scholarly impact, they are correlated with publication count, and, thus, also subject to the gender and potential ethnicity biases we identified in our analysis. The inevitable consequence is the emphasis on quantity over quality, and the reinforcement of existing prestige hierarchies, potentially further disadvantaging women and minoritized researchers, known as the ‘Matthew effect’ [41,60–63].

Our study revealed an increase in publication rates during the COVID-19 pandemic, particularly among women. This is contrary to other fields and faculties in other countries where women, especially Black women and mothers [64], were negatively affected by the pandemic in terms of publication productivity [65,66]. This finding may be explained by the fact that many researchers in our departments had a ‘key worker’ status during the pandemic, and thus had less caretaking responsibilities than researchers in other disciplines. However, this finding also highlights the importance of conducting studies in different fields where specific contexts affect gender bias differently.

Although we have identified biases in publications within SPH, the complete set of reasons causing this bias is not immediately apparent. There is reasonable evidence that gender bias may exist in scientific publishing [67]. While some studies show the chance of being published when submitting a paper to a journal is equal for all genders [48], Kern-Goldberger’s [67] and others’ studies demonstrate a difference in the acceptance rate based on perceived author. Holman *et al.* propose that this could partially be explained by journals inviting men to submit papers at double the rate of women [68]. However, the persistent gender gap suggests the issue extends beyond biases in the peer-review process. Recent evidence supports the view that gender gaps in publishing are driven more by disparities in submission rates than by overt bias in review or acceptance. For example, a 20 years audit of *Socio-Economic Review* found women consistently under-represented among authors, yet acceptance rates did not differ by gender once papers were submitted, indicating that the main gap lay in the number of submissions [69]. An internal analysis of approximately 215 000 submissions to *Nature* journals similarly reported that only approximately 17% of corresponding authors were women, but female-led papers were equally likely to be reviewed and accepted as those by men, with slightly higher acceptance when evaluated by mixed-gender reviewer panels [70]. Comparable findings from biomedical journals [71] reinforce that women’s under-representation arises largely at the submission stage, probably reflecting upstream structural and social factors rather than systematic bias during peer review.

Long-standing claims attribute productivity gaps to caretaking responsibilities (maternity leave and caring for children and ageing parents; [72]), with women having shorter career length [73] and higher drop-out rates [73,74]. However, the literature suggests a more complex picture that cannot be reduced to old-fashioned gender roles and responsibilities. Disparities in lifetime academic productivity can be traced to factors like unequal research funding [75] and under-representation on editorial boards [76]. Moreover, women disproportionally engage in teaching, community engagement and ‘academic housework" [38], possibly because they do not say ‘no’ [77]. Thus, evaluations ought to be more comprehensive to account for the various facets of activities researchers engage in, such that all the women’s ‘yes’s’ are valued. While ‘communal’ skills are more frequently mentioned in recommendation letters for promotion among women [31], such efforts should receive more attention as research activities in evaluations. Furthermore, women are more likely to experience imposter syndrome than men [78], which may lead to a lack of confidence in publishing. One limitation in interpreting these patterns is that our analysis may not fully capture career-stage effects: because promotion dates were unavailable, job categories were inferred from the most recent title. This assumption could obscure differences in productivity and recognition that arise during transitional career stages, which are particularly relevant to understanding gender disparities. Another limitation relates to the ‘survivorship bias’ since our data review the publication history of SPH researchers who remained affiliated to our institution until 2023 and does not account for researchers who left prior to this, potentially owing to inequity and pressures described in this study. It was not within the scope of this research to address the implications of this form of bias, but future studies would benefit from consideration of how to engage with individuals who have felt forced out of academia.

In SPH, there was a less clear trend of publication rates by ethnicity–there was an ethnicity effect only in women overall, and in men only when considering first/last author papers. However, with non-minoritized researchers having significantly more co-authors than minoritized researchers, this suggests that teamwork can be used more by researchers from non-minoritized groups, potentially contributing to their productivity as measured in terms of publication count. This finding must be interpreted with caution as we did not have ethnicity recorded and the number of researchers of other ethnicities is low, such that an effect may not be detectable. Researchers of minority groups may have fewer opportunities for collaboration as very rarely afforded the opportunity to do so. We suggest further research on this.

The absence of a significant difference in overall publication rates between researchers from minoritized and non-minoritized groups implies that additional systemic barriers hinder the promotion of individuals from minoritized groups into tenure-track positions. In part, longer acceptance delay, fewer citation counts and under-representation on editorial boards are possibly contributing to the hurdles experienced by researchers from minoritized groups (and women) [79,80]. Furthermore, our findings suggest that there may be barriers to co-authoring papers led by first or last authors from minoritized groups such that they benefit less from the work effort of bigger teams, and, thus, produce fewer publications. The finding is important to discuss further as the overall proportion of researchers from minoritized groups is significantly smaller compared to London’s ethnicity profile, where ICL is based [81]. This is consistent with the broader profile of academia in STEM, where there is under-representation of staff and students from minoritized groups [82]. Yet, the proportion of researchers from minoritized groups in our study is similar to the proportion of people from minoritized groups living in England and Wales [83]. There may also be intersection with other factors affecting academic promotion. For example, in North America, parental socioeconomic status has been shown to impact academic experiences [84] and accessibility to tenure-track positions, with tenure-track holders nearly twice as likely to have PhD-holding parents as their non-tenure PhD-holding peers [85].

To better understand why gender bias continues to exist in SPH, and to capture diverse insights and experiences which can inform a positive change in practice, we are running focus group discussions with representatives from all gender groups, ethnicity groups and job levels. We also plan to expand this analysis beyond SPH to the broader Faculty of Medicine to investigate the presence of broader systemic bias and if there are groups within ICL that SPH can learn from.

We propose a more proactive approach to monitoring publication rates and other types of research engagement activities in faculties. We appreciate that the issue of faculty diversity in higher education has gained significant attention in recent years, especially in the US [86], and the literature on this matter is growing. However, just as staff numbers are regularly analysed for the Athena Swan Charter, we advocate for routine, potentially automated, analyses of research engagement activities stratified by job category, ethnicity and gender. This monitoring should be assigned as a paid responsibility to a designated individual or team, rather than relying on volunteers. Notably, the methodology should be chosen with respect to the field, appreciating that different fields have distinct research cultures. For example, natural sciences tend to prioritize collaborative journal articles, while many humanities disciplines tend to value single-authored books or individual book chapters. This diversity challenges one-size-fits-all evaluation methods. Yet, our methodology and code may be used as a starting point for EDI in other settings. We also acknowledge that publication rates may correlate with grant funding, another area that has known disparities leading to differential attainment. We were unable to consider this as part of our analysis for this study, but formal analysis between funding success and publication rate may add further detail to the underlying causes and therefore solutions of the problem.

Identifying and acknowledging biases is a crucial first step towards EDI. Departmental analysis, as used to inform this Commentary, can confirm existing hypotheses and guide focused action, while also revealing areas where investment may not be necessary. Failing to recognize bias has detrimental effects, but falsely assuming sexism is widespread in academic science also carries significant costs. It can discourage women from pursuing careers in scientific academia and lead to resources being misspent on addressing non-existent bias [21].

Analyses and discussions as presented here are equally relevant for establishing more gender-balanced work environments in young, diverse institutions and retaining diverse talent in faculties with a long history of inequities. While some struggle with under-representation, others may see an over-representation of women. Ultimately, achieving equity requires continuous effort and a commitment to inclusivity across all genders and ethnicities in all job levels.

It is our view that when addressing barriers to academia and promotion, we must ensure first that there is equity in opportunities to publish and secondly that research contributions other than publications and other work activities in which researchers may engage more are equally valued and recognized. Continued efforts should be made to achieve an inclusive research culture by examining potential biases in research funding, mentorship and promotion processes, to support equity and retain a diverse generation of future researchers. We conclude this work with a set of recommendations for others to consider in evaluating EDI in their department (box 1).

**Box 1. Institutional recommendations to foster equity and inclusion:**

**1. Regular monitoring and analysis:** implement routine, automated, analyses of research engagement activities to account for nuances in productivity measures, stratified by job category, ethnicity and gender using empirical data (i.e. ideally not relying on probabilistically assigned ethnicity information). This monitoring should be an assigned, paid responsibility (e.g. individuals at EDI Centres), not reliant on volunteers, and should be reviewed regularly at departmental meetings (e.g. Athena Swan and Race Equality Charter committee meetings, senior management meetings, people and culture committees);

**2. Mixed-methods approach:** use both qualitative and quantitative data for comprehensive evaluations of barriers and facilitators to publishing, career progression and maintaining a diverse and accepting working environment, drawing on the strengths of each approach;

**3. What works:** determining what institutional environments and policies reduce publication bias to draw up a comprehensive, clear list of recommendations that departments can act on;

**4. Early intervention in young institutions:** proactively establish equitable work environments and policies in newly founded institutions to prevent the entrenchment of biases; and

**5. Acknowledge and address biases:** openly identify and acknowledge biases as well as successes within the system and commit to focused action to address and maintain them.

## Data Availability

The code required to rerun the analysis in this study is available at https://github.com/paulachristen/publication_bias_script. Supplementary material is available online [87].

## Footnotes

^1^
The analysis underlying this comment was supported by a larger cohort of researchers in our department, who all contributed their perspectives and ideas at a Hackathon in June 2023.
